# Reversing resistance to antiandrogens with a histone deacetylase inhibitor

**DOI:** 10.18632/oncotarget.26464

**Published:** 2018-12-18

**Authors:** Anna C. Ferrari

**Affiliations:** Anna C. Ferrari: Visiting Professor, Division of Medical Oncology, Icahn School of Medicine at Mount Sinai, One Gustave L. Levy Place, New York, NY, USA

**Keywords:** resistance reversion, antiandrogen, histone deacetylase inhibitor, castration resistant, prostate cancer

Overexpression of the androgen receptor (AR) mRNA and protein is the most prevalent alteration in castration-resistant prostate cancer (CRPC) that is due to amplification of the AR gene and/or an upstream enhancer in a non-coding region in up to 81% of cases [1]. AR overexpression is sufficient for the transition from hormone-sensitive to lethal CRPC by increasing sensitivity to low androgen levels and estrogens [2] and by increasing, pathogenic ligand-independent AR mRNA splice variants (ARSv) [3].

Current treatment strategies aim to control AR-driven CRPC by blocking the AR ligand-binding domain (LBD), decreasing alternative sources of ligands or by employing an antagonist that alters AR conformation. However, since these measures do not affect AR mRNA synthesis, increasing levels of AR protein have been shown to shift the abundance and ratios of co-activators over repressors assembled on the promoters of AR target genes and decrease bicalutamide binding, generating resistance [2, 4]. The synthesis of ARSv7 further contributes to antiandrogen resistance, since it lacks the targeted LBD but retains a distinct mitosis-dependent transcriptome program that promotes CRPC progression [3]. Thus, reducing AR mRNA levels may be critical for prolonging the clinical benefits of AR antagonists by sustaining their efficacy and avoiding the generation of ARSv7.

Generally, treatment strategies aiming to suppress aberrant gene transcription have met limited success. However, CRPC with its essential component of resistance development to antiandrogen agents after prolonged treatment, that result in strong dependence on AR overexpression, independently or through cross-talk with associated oncogenic pathways, may be unusually susceptible to this strategy. In particular, broadly-targeted epigenetic mechanisms affecting chromatin acetylation status by the opposing actions of histone acetylases (HATs) and deacetylases (HDACs) are critically involved in fine-tuning regulation of AR mRNA transcription and splicing, as well as the transcriptional activation of AR protein target genes [5]. Although the molecular details of how these opposing enzymes affect the AR transcriptional processes is incompletely understood, a consistent paradoxical finding in CRPC models is that HDACs, which are classically a key component of the repressor complex, are required for AR mRNA transcription and protein stability and for transcriptional activation of AR target genes [6]. Accordingly, pan HDAC inhibitors (HDACIs), such as Panobinostat, uniformly reduce AR mRNA levels, including ARSv7 in CRPC models [7]. Consequently, AR protein synthesis and levels are also diminished by HDACIs, although there are conflicting data about the contribution of cytoplasmic AR protein degradation via HDACI-induced acetylation of the transporter protein HSP90 [6]. HDACIs also reduce the level of important AR target genes, including PSA and KLK2, but, notably, the effect is more selective and requires a higher concentration of the HDACI. Biologically, HDACI molecular effects are accompanied by reduced cell proliferation *in vitro* and tumor growth *in vivo* in all CRPC model systems [6]. Unfortunately, these preclinical observations did not translate into beneficial clinical activity of HDACI monotherapy in CRPC, possibly due to not achieving sufficiently high and sustained drug concentrations.

An alternative perspective is that HDACIs might be more effective in overcoming the prevalent acquired resistance to anti-androgenic agents in CRPC compared to their direct anti-tumor effects.

We demonstrated in preclinical experiments with CRPC culture/xenograft models that overexpress AR and ARSv7 that there is synergistic inhibition of cell growth by Panobinostat in combination with the antiandrogen bicalutamide in cells resistant to bicalutamide [7, 8]. Notably, an approximately 2-fold higher concentration of Panobinostat in the combination was required to further produce apoptosis [8]. Based on these preclinical results, we designed a phase I/II study in CRPC patients resistant to one or more first generation antiandrogens: treatment with bicalutamide 50 mg daily continuously together with Panobinostat on an intermittent schedule. In Phase I, the MTD was not reached in the highest dose cohort of Panobinostat, 40 mg PO thrice weekly × 3 weeks. The randomized phase II evaluated efficacy and tolerability of the combination at a high (40 mg) and low (20 mg) dose of Panobinostat thrice weekly for 2 of 3 weeks. The results showed that both dose levels of the combination exceeded the protocol-specified 35% probability of remaining radiographic progression-free (rPF) at 36 weeks (47.5%; 38.5%). However, the 40 mg but not the 20 mg dose-treated patients exceeded expectations for median time to radiographic progression (rP; 33.9 and 10 weeks) and time from PSA progression to rP (24 and 5.9 weeks). Toxicity G1-2 was similar to single-agent Panobinostat in both arms. G3 toxicity prevailed at the high dose and caused early withdrawals, but it was controlled with dose reductions [7]. These results provide evidence that, in combination with bicalutamide, Panobinostat had a beneficial clinical effect in extending rPF survival at the higher 40 mg dose, which was associated with greater but manageable toxicity.

In contrast to Panobinostat monotherapy, these results are consistent with a model in which rewriting the epigenetic code by a HDACI resensitizes the CRPC to the antiandrogen they became resistant to [9] by suppressing AR mRNA and protein synthesis, reducing AR protein to a level that restores the antiandrogen binding and antagonistic function over oncogenic pathways [2, 4]. Since the epigenetic changes induced by the HDACI may be reversible, the frequent intermittent exposure to Panobinostat in the presence of bicalutamide may have been critical for the resensitization and maintenance of bicalutamide antagonistic function. Another possibility, given the much higher incidence of early rP on 20 mg is that a starting dose of 40 mg Panobinostat in combination with bicalutamide had an early direct anti-tumor effect, analogous to our afore-mentioned preclinical observation that a 2-fold increase in the cytostatic concentration of Panobinostat was cytocidal [8]. Also, since the high-dose combination was only effective for a limited time, another consideration could be to introduce the HDACI earlier during the response to antiandrogen to prevent epigenomic and possibly genomic resistance mechanisms [9, 10]. The promising results of our trial supports implementation of a successor trial in equivalent CRPC patients resistant to the more powerful antiandrogen enzalutamide, which also works by binding to the AR LBD and develops resistance linked to AR overexpression and ARSv7.
